# Supplementary figures and images for: Causal debiasing for unknown bias in histopathology—A colon cancer use case
Source: PLoS One. 2024 Nov 22;19(11):e0303415. doi: 10.1371/journal.pone.0303415 (PMC11584097; doi:10.1371/journal.pone.0303415)

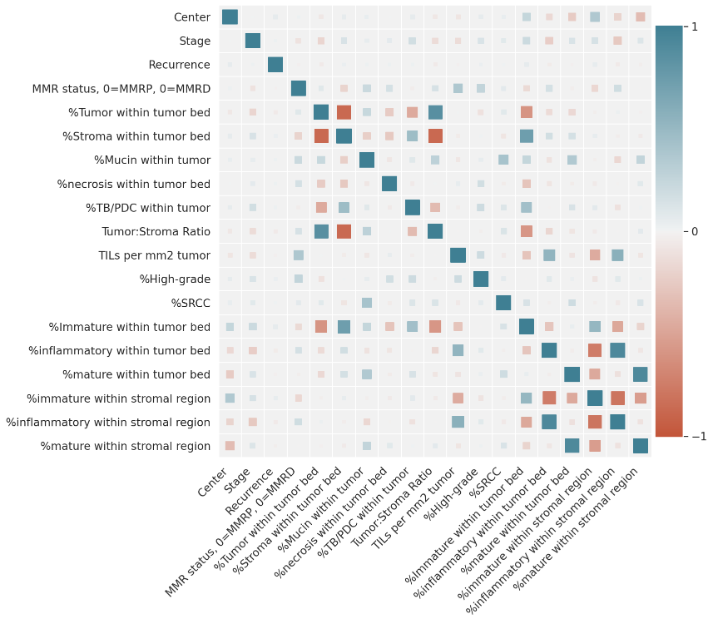

Supplement: S1 Data — (ZIP) [file pone.0303415.s001.zip › rlcorrea_ColonCausal_PlosOne-2/FeatureCorrelation_stage_centered.png]

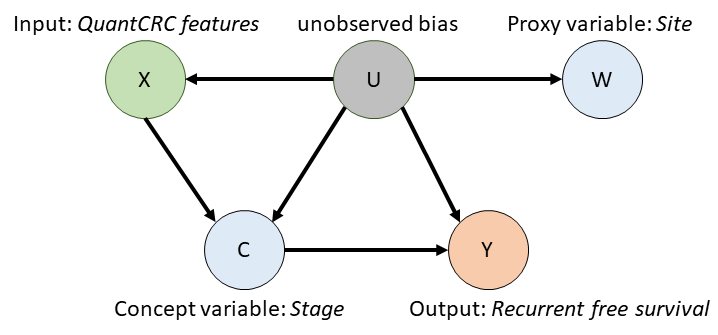

Supplement: S1 Data — (ZIP) [file pone.0303415.s001.zip › rlcorrea_ColonCausal_PlosOne-2/causal_diagram.png]

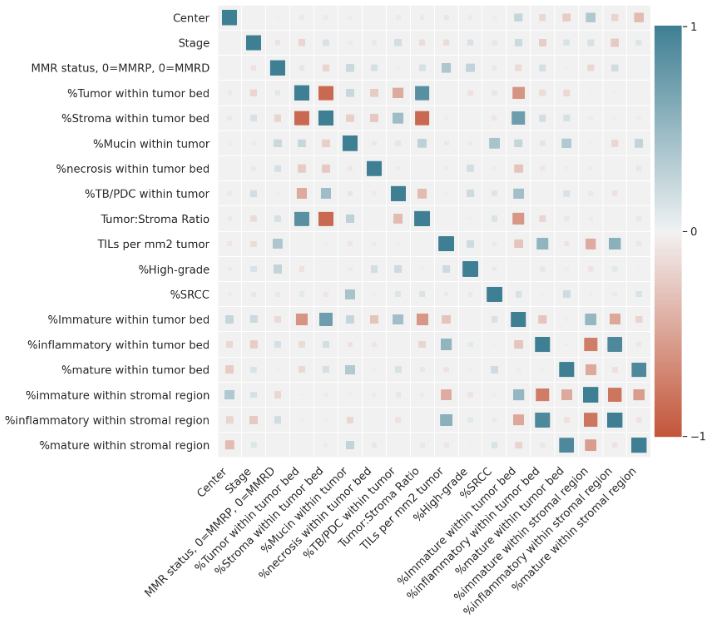

Supplement: S1 Data — (ZIP) [file pone.0303415.s001.zip › rlcorrea_ColonCausal_PlosOne-2/FeatureCorrelation.png]

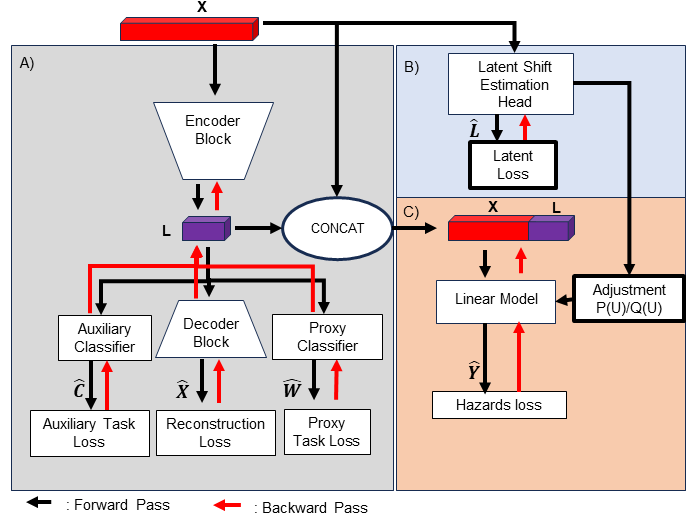

Supplement: S1 Data — (ZIP) [file pone.0303415.s001.zip › rlcorrea_ColonCausal_PlosOne-2/fair_risk_model.png]

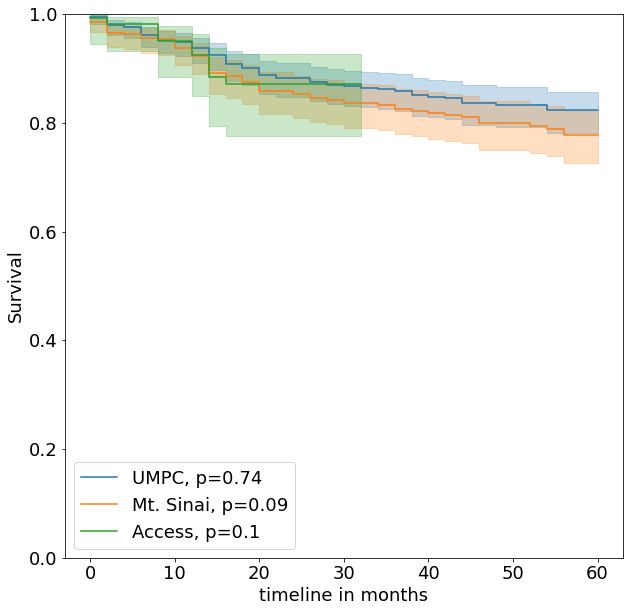

Supplement: S1 Data — (ZIP) [file pone.0303415.s001.zip › rlcorrea_ColonCausal_PlosOne-2/ExternalSurvival.png]

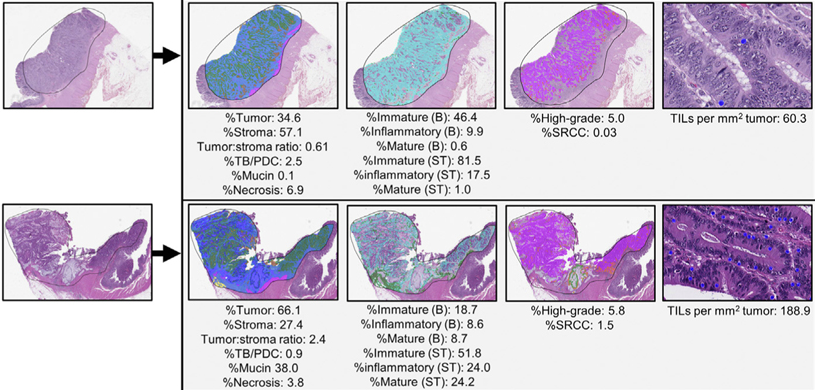

Supplement: S1 Data — (ZIP) [file pone.0303415.s001.zip › rlcorrea_ColonCausal_PlosOne-2/QuantitativeFeatures.PNG]

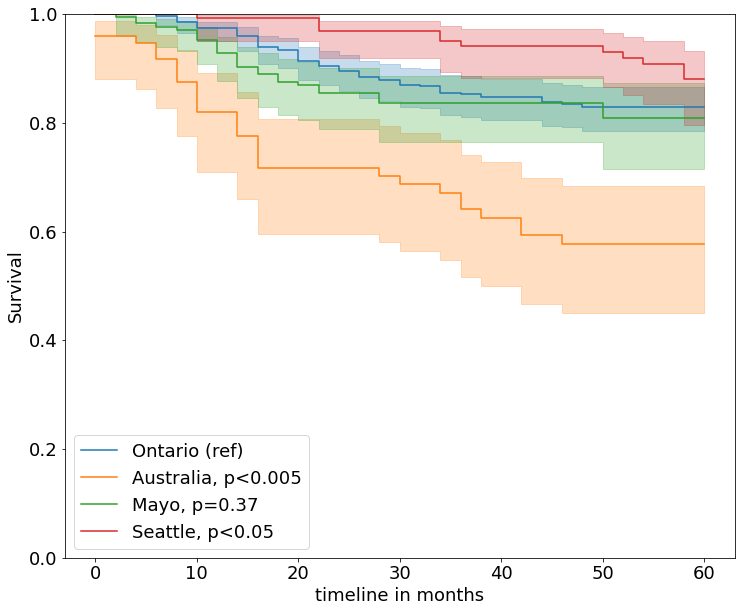

Supplement: S1 Data — (ZIP) [file pone.0303415.s001.zip › rlcorrea_ColonCausal_PlosOne-2/internalSurvival.png]

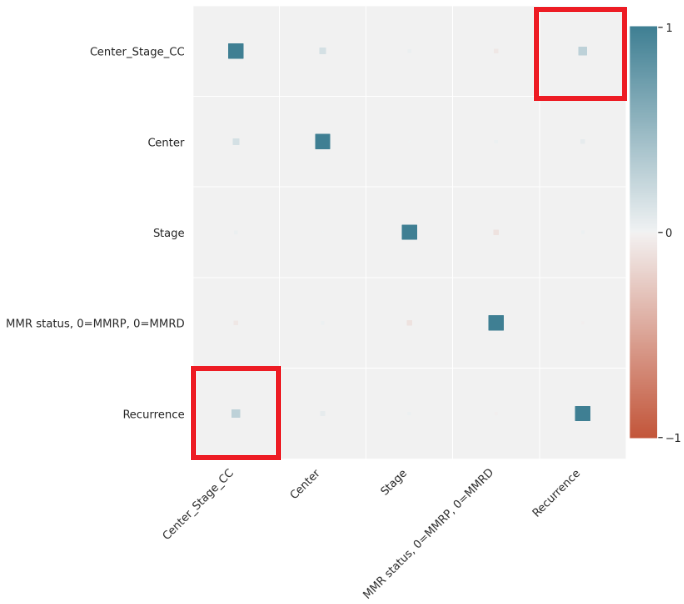

Supplement: S1 Data — (ZIP) [file pone.0303415.s001.zip › rlcorrea_ColonCausal_PlosOne-2/Correlation_stage_centered.png]

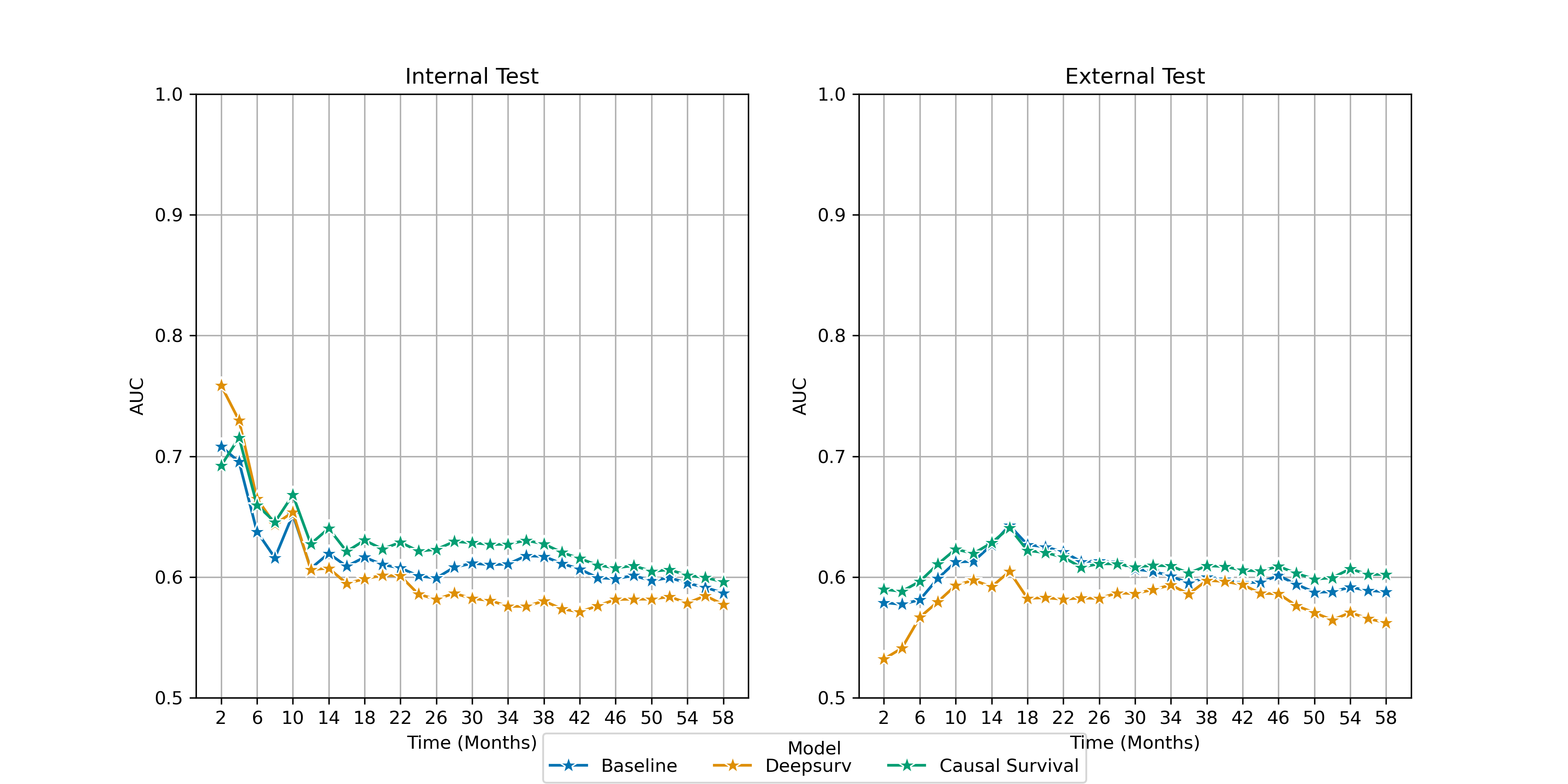

Supplement: S1 Data — (ZIP) [file pone.0303415.s001.zip › rlcorrea_ColonCausal_PlosOne-2/time_based_auc_none.png]
